# The Tuberculous Granuloma: An Unsuccessful Host Defence Mechanism Providing a Safety Shelter for the Bacteria?

**DOI:** 10.1155/2012/139127

**Published:** 2012-07-03

**Authors:** Mayra Silva Miranda, Adrien Breiman, Sophie Allain, Florence Deknuydt, Frederic Altare

**Affiliations:** INSERM U892, CNRS UMR 6299, Université de Nantes, 44007 Nantes Cedex 1, France

## Abstract

One of the main features of the immune response to *M. Tuberculosis* is the formation of an organized structure called granuloma. It consists mainly in the recruitment at the infectious stage of macrophages, highly differentiated cells such as multinucleated giant cells, epithelioid cells and Foamy cells, all these cells being surrounded by a rim of lymphocytes. Although in the first instance the granuloma acts to constrain the infection, some bacilli can actually survive inside these structures for a long time in a dormant state. For some reasons, which are still unclear, the bacilli will reactivate in 10% of the latently infected individuals, escape the granuloma and spread throughout the body, thus giving rise to clinical disease, and are finally disseminated throughout the environment. In this review we examine the process leading to the formation of the granulomatous structures and the different cell types that have been shown to be part of this inflammatory reaction. We also discuss the different *in vivo* and *in vitro* models available to study this fascinating immune structure.

## 1. Introduction

Almost 20 people develop tuberculosis and four people die from the disease every minute, somewhere in the world [1]. Tuberculosis thus remains a major disease in terms of mortality and morbidity. Almost one-third of the human population is infected with the bacillus, but less than 10% of those infected go on to develop the disease. In the other infected individuals, of whom there are thought to be two billion worldwide, the disease remains in a latent state. These individuals serve as a reservoir of the bacterium and, if they become immunodepressed, they may present a reactivation of the disease, leading to the spreading of the infection. More detailed studies of tuberculosis pathogenesis have thus become essential, to find a way to eradicate this disease once and for all. Considerable efforts have been focused on the development of an effective vaccine and new treatments, but we also need a good study model. This will enable us to improve our understanding of the physiology of the disease, making it possible to find new ways of combating tuberculosis and providing us with greater knowledge of this host-parasite relationship.

The prognosis of the disease depends on the ability of the host to eliminate the bacillus. The respiratory route is the principal route of infection. The disease starts when droplets from actively or latently infected individuals reach the respiratory tract of healthy individuals. These droplets contain a small number of bacilli that enter the lung, where they infect primarily alveolar macrophages, type 2 pneumocytes, and polymorphonuclear neutrophils (PMNs) [2]. In most individuals, the infection poses few problems to health, because the bacteria have developed an ability to live in balance with immune responses. This bacillus is an enormously successful human pathogen that can infect its host for decades without causing clinical disease, with reactivation occurring only when the immunity of the host is compromised.

Many studies have suggested mechanisms for the initial events in the lung, but most were based on experiments carried out *in  vitro* with cell lines. Such conditions do not provide information about the real sequence of events taking place when the bacilli gain access to the lung. A knowledge of the whole process is required if we are to determine why some individuals develop the disease whereas the disease remains latent in others. The immune system undoubtedly plays a major role, but we do not currently have an appropriate tuberculosis model for its investigation.

**Figure 1:**
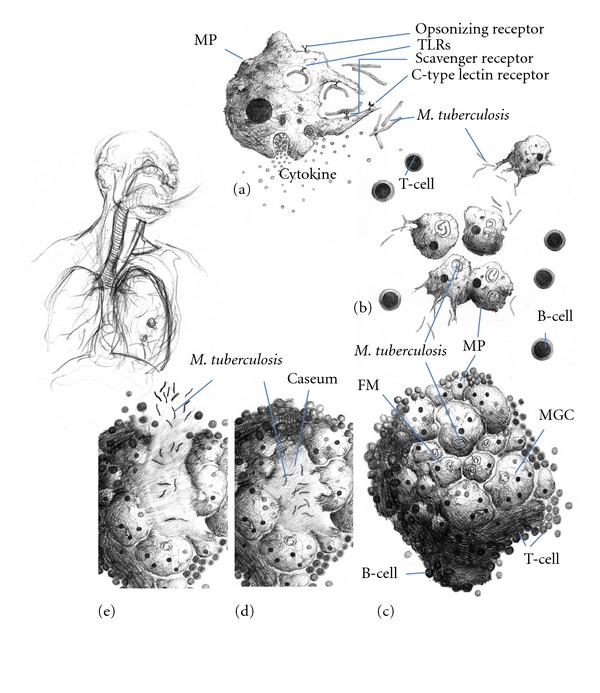
Formation and maturation of lung tuberculous granulomas. Following inhalation of contaminated aerosols, *M*. *Tuberculosis *moves to the lower respiratory tract where it is recognized by alveolar macrophages. This recognition is mediated by a set of surface receptors (see text), which drive the uptake of the bacteria and trigger innate immune signalling pathways leading to the production of various chemokines and cytokines (a). Epithelial cells and neutrophils can also produce chemokines in response to bacterial products (not represented). This promotes recruitment of other immune cells (more macrophages, dendritic cells, and lymphocytes) to the infection site (b). They organise in a spherical structure with infected macrophages in the middle surrounded by various categories of lymphocytes (mainly CD4^+^, CD8^+^, and *γ*/*δ* T cells). Macrophages (MP) can fuse to form MGCs or differentiate into lipid-rich foamy cells (FM). B lymphocytes tend to aggregate in follicular-type structures adjacent to the granuloma ((c), see text for details). The bacteria can survive for decades inside the granuloma in a latent state. Due to some environmental (e.g., HIV infection, malnutrition etc.) or genetic factors, the bacteria will reactivate and provoke the death of the infected macrophages. A necrotic zone (called *caseum* due to its milky appearance) will develop in the centre of the granuloma (d). Ultimately the structure will disintegrate allowing exit of the bacteria, which will spread in other parts of the lungs and more lesions will be formed. Infection will also be transmitted to other individuals due to release of the infected droplets by coughing (e).

We know that the infected alveolar macrophages in the lung release various cytokines to recruit different populations of cells, including more macrophages, to the infection site. Dendritic cells are important because they present antigens to T cells in the lymph nodes, in which a T-cell response can subsequently be developed. These signalling events lead to the formation of a granuloma, the hallmark of tuberculosis. This structure is developed by the host to contain the infection and eliminate the bacteria. However, the bacteria persist in a latent state within the granuloma, often for decades. They subsequently reactivate in 10% of the latently infected individuals. The death of the infected cells causes the formation of a necrotic zone in the centre of the granuloma, which eventually disintegrates, releasing the bacteria into the lung, and thence into the environment (see Figure 1).

## 2. Granuloma Formation

The granuloma, the hallmark of tuberculous disease, creates an immune microenvironment in which the infection can be controlled. However, it also provides the mycobacterium with a niche in which it can survive, modulating the immune response to ensure its survival without damage over long periods of time [3, 4]. One of the most important factors required for the establishment of infection is a balance between the proinflammatory and anti-inflammatory cytokines produced to reduce or control bacterial proliferation. TNF-*α* and IFN-*γ* are particularly important in promoting the formation and function of the granuloma, whereas IL-10 is one of the main negative regulators of the response [5–7] (see Table 2).

The granuloma contains mostly blood-derived macrophages, epithelioid cells (differentiated macrophages) and multinucleated giant cells (also known as Langhans giant cells), surrounded by T lymphocytes [8, 9]. Caseous granulomas are typical of tuberculosis. These structures are formed by epithelioid macrophages surrounding a cellular necrotic region with a rim of lymphocytes of the T- and B-cell types. Other types of granuloma include nonnecrotising granulomas, which consist primarily of macrophages with a few lymphocytes, necrotic neutrophilic granulomas, and completely fibrotic granulomas [10, 11].

Some lymphoid clusters organised similarly to the follicular centres of lymph nodes are also associated with granulomas (see Section 4.2.5). These structures seem to be sufficient for T-cell priming, and secondary lymphoid organs (such as the lymph nodes and spleen) do not seem to be essential for an effective antimycobacterial response [12]. Recent studies of zebrafish infected with *M. marinum* have suggested that the innate response may contribute to the control of mycobacterial growth [13]. However, T cells are activated following exposure to *M. tuberculosis *(Mtb) and are an essential component of the protective response [14]. Studies using live imaging of zebra-fish as well as some recent studies of hepatic granuloma formation in mice using intravital 2-photon microscopy have shed light on the dynamic events in the development of a granuloma [13, 15–18]. Egen et al. found that the granuloma starts by aggregation of different subsets of cells from the macrophages/monocytes lineage and observe marked morphological changes of those cells. T cells were then rapidly recruited to the forming granuloma. The macrophages were found to remain relatively static in the structure, whereas T cells were highly motile, although they were retained in the granuloma most likely through interaction with the macrophages [17]. Noteworthy, the situation was slightly different in the zebrafish where macrophages were seen to migrate out of the granuloma at different stages [13, 16]. Interestingly, Egen et al. also found that mycobacteria-specific T cells were hardly more arrested than T cells of other specified during their migration through the lesion and that their cytokine production was moderate, suggesting that presentation and/or recognition of antigen is limited inside the granuloma [18].

Many different chemokines are involved in granuloma formation (see Table 2). Some are produced by the epithelial cells of the respiratory tract, and others are produced by the immune cells themselves. In particular, the chemokines binding to the CCR2 receptor (CCL2/MCP-1, CCL12, and CCL13) are important for the early recruitment of macrophages. Osteopontin, which is produced by macrophages and lymphocytes, promotes the adhesion and recruitment of these cells (reviewed in [19]).

CCL19 and, possibly, CCL21 are involved in the recruitment and priming of IFN-*γ*-producing T cells. CXCL13 is involved in B-cell recruitment and the formation of follicular structures [20]. The expression of the CC and CXC chemokines is deregulated at the transcriptional level in TNF-deficient mice, and the lack of these chemokines prevents the recruitment of macrophages and CD4^+^ T cells, accounting for the critical role of TNF-*α* in granuloma formation [21].

## 3. Models Used to Study *M. tuberculosis* ****Infection and the Granulomatous Response

Animal models are often used to study granulomatous structures. These models re-produce many of the processes occurring in humans, although differences are frequently observed. It is difficult to study biopsy samples from the lung, to which access is often limited. This has led to the widespread use of animal models, which have been improved over the years to reproduce more closely the progression of the disease observed in humans. A large number of mouse models of infection have been generated, but the most relevant is probably that based on the intranasal infection route, because this is the route involved in natural infections in humans [11]. Nevertheless, all the available mouse models have generated valuable information, increasing our understanding of the host-pathogen relationship. Mice develop an acute, rather than chronic, infection with Mtb and the granuloma in the lung lacks the structured and organised appearance of human granulomas.

However, murine granuloma models that more closely resemble granulomas in humans have recently been described. Nos 2^−/−^ mice infected with *M. tuberculosis* develop granulomas similar to those of humans, in the lung [22]. In addition, Harper et al. and Driver et al. recently reported that C3HeB/FeJ mice develop necrotic lesions in response to *M. tuberculosis* infection [23, 24]. This is currently the only relevant animal model for studying necrosis. Harper et al. also showed that positron emission tomography (PET) techniques could be used to study the necrotic lesion in mice. By infecting C3HeB/FeJ mice and following the infection with PET technology, they demonstrated that the granulomas induced in these mice were hypoxic, whereas those induced in BALB/c mice were not. They also reported the overexpression of certain hypoxic genes in these mice [23]. In addition, Driver et al. found that, after antimycobacterial treatment, higher numbers of drug-resistant bacteria were isolated from C3HeB/FeJ mice than from BALB/c mice [24]. These two recent mice models thus greatly improve the relevance of the mouse as an *in vivo* model to study the pathophysiology of the natural human infection.

Rabbits and guinea pigs, which form granulomas largely similar to those in humans, have also been studied [25]. These models, although very nicely mimicking the human situation, are still poorly used, mainly due to the scarcity of cell culture reagents. Davis et al. have described an unusual new model of mycobacterial infection in zebrafish embryos. Mycobacterial granuloma formation and the replication of mycobacteria can be studied in real time in this model, due to the transparent body of the larva [13]. The zebrafish embryo has provided a unique tool for visualising subtle features of host-pathogen interactions and monitoring the progression of the pathological consequences of infection in a living organism. Nonhuman primates (NHPs), who develop a disease similar to that in humans, have also been studied. The NHP model generates results similar to those for humans, and samples can be taken at various time points. This model has the advantage of controling over the strain, dose and timing of infection in the animals, these factors being very difficult to assess in humans [26, 27].

Computer models have also recently been developed, to describe or to make predictions based on general information about tuberculosis disease [7, 28–30]. Magombedze and Mulder, for example, have recently developed a dynamic theoretical model of Mtb latency. This model considers changes in gene expression and in all the factors involved in active tuberculosis [31]. These models are particularly useful if we can supply them with experimental observations, but they also make it possible to study aspects that cannot be investigated in the laboratory.

Zucchi et al. established a model of tuberculosis in the central nervous system (CNS), in which the injection of mycobacteria into the cerebellum induces granuloma formation. This model is useful for studies of Tb meningitis and of other types of extrapulmonary tuberculosis. The principal drawback of this model is the infection route, which differs radically from the natural route of infection. However, as a model of granuloma formation, it is a useful tool for studying the physiopathology of brain infection and pathogen/host dynamics [32].

*In  vitro* models have also been developed for the investigation of Mtb infection in single-cell lines, mostly derived from macrophages. In this context, we described an *in  vitro* granuloma model in 2004. This model consists of human blood cells infected with mycobacteria or treated with mycobacterial antigens, resulting in the formation of a typical epithelioid granuloma with morphological characteristics and cellular differentiation levels similar to those of natural granulomas [9, 33]. Using this *in vitro* human model, important information have been achieved about granuloma cells differentiation, as well as about cellular interactions and cell/bacteria interplay within granulomatous structures [9, 33–35]. This model can be used to study both active infection and latent states (see Table 1).

## 4. The Principal Cell Populations Involved in the Granulomatous Response

### 4.1. Monocyte-Derived Cells

#### 4.1.1. Macrophages

Most of the macrophages involved in granuloma formation are epithelioid macrophages. These cells are activated and have an abundant cytoplasm. After initial infection, the macrophages (monocytes when immature) migrate to the infection site from the blood. They have various innate immune receptors in their membranes, allowing them to recognise, bacteria, take them up by phagocytosis, and secrete various cytokines. These receptors belong to four main classes: opsonizing receptors (e.g., Fc*γ*R and complement receptors), scavenger receptors (e.g., CD36 and MARCO), C-type lectin receptors (e.g., mannose receptor, dectin-1, dectin-2, and DC-SIGN) and innate immune sensors (e.g., TLRs and NODs). The most studied receptors in tuberculosis granulomas are TLR2, TLR4, TLR9, mincle, dectin-1, DC-SIGN, mannose receptor, complement receptors, and NOD2 [6, 11, 36–43].

As pointed out above, macrophages are the principal cells found in granulomas, but not all the macrophages in the granuloma are infected. The uninfected cells seem to help to contain infection and contribute to cytokine secretion. Some studies have suggested that there are two kinds of macrophages: classically activated macrophages (CAM), which differentiate in response to cytokine signals, or alternatively activated macrophages (AAM). CAMs, which are induced by the secretion of Th1 cytokines, are able to kill bacteria. In murine models, these cells produce iNOS. This enzyme catalyses the synthesis of nitric oxide (NO), a potent antimicrobial compound. The production of iNOs is strongly induced by IFN-*γ*. Conversely, AAMs are induced by Th2 cytokines (IL-4, IL-13). These cells produce anti-inflammatory cytokines (IL-10, TGF-*β*) and arginase, which compete with iNOS for the use of arginine as a substrate [44].

Recent data from mouse models suggest that macrophages of the AAM type are also found in the tuberculous granuloma. The TLR signalling triggered by mycobacteria may lead to the induction of arginase production by macrophages, through the MyD88-dependent production of IL-10, IL-6, and granulocyte colony-stimulating factor (G-CSF). Switching off arginase expression has been shown to be beneficial for host survival [42, 45]. The presence of both types of cells in the granuloma may be required to maintain a balance between pro- and anti-inflammatory cytokines. However, it may also allow the bacteria to survive in infected macrophages by promoting the production of arginase to prevent NO synthesis.

#### 4.1.2. Multinucleated Cells

Macrophages may also fuse to generate multinucleated Langhans giant cells (MGCs), which are characteristic of tuberculosis. The ontogeny of these cells has only recently been described [46]. Our team recently reported that the fusion process could be triggered in a TLR2-dependent cell activation by mycobacterial lipomannan (but not lipoarabinomannan), and that this process was dependent on a ß1 integrin/ADAM9 pathway [47]. Another very recent study showed that the coculture of macrophages with activated T cells can lead to MGC formation through CD40/CD40L interaction and IFN-*γ* secretion [48]. MGCs are found only in the granulomas induced by Mtb. Granulomas induced by weakly virulent mycobacteria may contain small-multinucleated cells (MCs), but these cells never differentiate into MGCs. MGCs have lost the ability to take up bacteria, because they no longer express the phagocytic receptors (mannose receptor and CD11b). However, they seem to have retained the ability to present antigens [33]. The loss of the phagocytosis capacity of MGCs suggests a possible role in a bacterial escape strategy driving the fusion of macrophages to form MGCs.

#### 4.1.3. Monocyte-Derived Dendritic Cells (mDCs)

mDCs have been found in the granulomas of tuberculosis patients [49] and mice [50]. They are found mostly towards the periphery of the lesion and contain fewer bacteria than macrophages. They are detected in the granuloma at early stages, subsequently moving to the draining lymph nodes, where they educate the T-cell response. Their antigen presentation function is impaired by the infection, although they continue to produce large amounts of MHC-II and costimulatory molecules [51]. Using fluorescently labelled DCs in a mouse model, Schreiber and coworkers observed a high exchange rate (one-third within a week) of inflammatory DCs within chronic stage granulomas, suggesting intense immune surveillance [52]. DCs are not very effective at killing *Mtb, *but they do keep the bacteria in a nonreplicating state. DCs containing live mycobacteria may stimulate T cells more effectively or may be used by pathogens as a vehicle for more efficient spreading [53, 54].

#### 4.1.4. Foamy Macrophages

Foamy macrophages are also classically found in human tuberculous granulomas. Their foamy appearance results from the accumulation of intracellular lipids within lipid bodies (LB) or droplets. The differentiation of macrophages into foamy cells can be triggered *in vitro* by infection with Mtb or by treatment with Mtb-specific envelope compounds, such as oxygenated mycolic acids [34]. TLR2 signalling has been implicated in this differentiation process in mice [55]. Ordway et al. also reported that murine foamy cells expressed a DC-type profile of surface markers (Dec205^+^ CD11b^+^ CD11c^high^ and CD40^high^ MHCII^high^), which may suggest some shared differentiation steps with DC [56].

Foamy cells are found within granulomatous structures in both animal and human models. We have shown that foamy macrophages have lost their phagocytic and bactericidal activities and that they allow Mtb persistence in a dormant state [34]. It is generally assumed that the lipids present in these cells can serve as a source of nutrients for the bacteria. The molecular links between these cells and bacterial latency in the tuberculosis granuloma, are still under study. To date, the *in vitro* culture of infected foamy macrophages constitutes the first physiological model of dormancy to be described, and as such, it could be very useful for the testing of candidate drugs active at this stage [34, 35]. In addition to being induced by *Mycobacterium tuberculosis*, foamy macrophages are also found in leprosy patients and in *M. avium*-infected AIDS patients [57, 58].

#### 4.1.5. Neutrophils

Neutrophils are also involved in the granulomatous response. These cells have been described as the first line of defence against tuberculosis. They are activated directly by Mtb products, such as lipoarabinomannan (LAM) in particular [59, 60]. The intranasal injection of LAM is sufficient to promote the influx of neutrophils into the lung and an IL-1-dependent inflammatory response in mice [61]. Neutrophils help to kill bacteria, and to initiate the inflammatory process, through the secretion of chemokines, such as MCP-1 and IL-8, to recruit leukocytes, and to organise the granuloma, through the secretion of CXCR3 chemokines (such as MIG, RANTES, and MCP-1) [59, 62]. Mycobacteria also seem to induce the production of oxygen radicals by neutrophils [60]. However, once the granuloma is established, neutrophils do not seem to play an important role in humans, returning to the infection site only when the granuloma starts to become necrotic (bacterial dissemination). By contrast, they are present in much larger numbers in mouse granulomas. These cells have been shown to be involved in the resistance of mice to *M. avium *infection [63]. They may act through the secretion of chemokines, but the precise role or mechanism of action of these cells in granuloma necrosis remains unclear.

### 4.2. T Lymphocytes

T lymphocytes account for 15 to 50% of the leukocytes in mouse granulomas. About 60–70% of the T cells present are CD4^+^, 15–30% are CD8^+^ *α*/*β* T cells, and there are also about 2%  *γ*/*δ* T cells [64]. Other minor subsets, such as NKT cells, are also present.

#### 4.2.1. T CD4^+^ Cells: Th1, Th17, and Treg Cells

The essential role of CD4^+^ T cells in the control of mycobacterial infection has been highlighted by many studies in knockout mice [65–67]. In MHC II^−/−^ and CD4^−/−^ mice, granuloma formation occurs about one week later than in wild-type mice. The lesions are less organised and their function is impaired, as they fail to control bacterial growth despite the macrophages displaying normal NO synthase expression [66]. Mice with defects in TCR recombination (RAG^−/−^) have a very poor granulomatous response to BCG infection, for example. However, this response can be restored to wild-type levels by the adoptive transfer of CD4^+^ T cells. In natural conditions, the CD4^+^ T cells of the granuloma have a diverse TCR repertoire, but reconstitution with a monoclonal population of CD4^+^ T cells is sufficient to restore granuloma formation [67].

Ordway et al. depleted mice of CD4^+^ T cells at different stages of infection by Mtb and showed that this caused disorganisation of the granulomatous lesion at all stages [68]. Deletion experiments in the *in vitro* model of human granuloma have suggested that CD4^+^ cells constitute the only T-cell population absolutely critical for granuloma formation (Allain et al., unpublished).

APC stimulates CD4^+^ T cells via TCR engagement, together with CD40-CD40L interaction and the production of IL-12. This leads to Th1 polarisation and the strong production of IFN-*γ*. Mice lacking MHC II or CD4 produce smaller amounts of IFN-*γ* (and, consequently, also of IL-12) in the early phase of the infection, but the concentration of this cytokine reaches normal levels after three weeks, due to compensation by CD8^+^ T cells. However, the early CD4^+^ T cell-dependent burst of IFN-*γ* production seems to be critical for the effective control of infection [66]. Similarly, the elimination of CD4^+^ T cells from Mtb-infected mice reduces Th1 cytokine secretion by a factor of 10 [68].

A study of CD40L^−/−^ mice showed that engagement of the costimulatory receptor was also required for the efficient recruitment of CD4^+^ T cells to granulomatous lesions. By contrast, CD28-mediated costimulation is not required, despite the smaller numbers of splenic CD4^+^ T cells and the lower level of activation of these cells in CD28^−/−^ mice. Some granulomas are formed in CD40L^−/−^ mice, but they fail to control bacterial growth effectively, resulting in a phenotype similar to that of IFN-*γ*-deficient mice [67].

A genetic syndrome called Mendelian susceptibility to mycobacterial infection has been described in humans. It is characterized by disseminated infections after vaccination with BCG or contact with nonvirulent mycobacteria (e.g., *M. avium*) and is linked to defects in IFN-*γ* or IL-12/IL-23 signalling (IL-23 is an IL-12-like cytokine; both make use of the p40 subunit and the IL12-Rß1 chain). Patients with complete IFN-*γ*R deficiency have a highly modified granulomatous response to BCG vaccination. Ill-defined, multibacillary “lepromatous-like” granulomas are formed, with a cell composition different from that of classical tuberculoid granulomas (fewer lymphocytes and giant cells, more granulocytes, and no necrosis). The formation of granulomas of this type is associated with a poor prognosis. By contrast, patients with partial IFN-*γ*R deficiency, who deal much better with the infection, produce mostly well-circumscribed paucibacillary granulomas. Patients with complete IL-12p40 or IL-12Rß1 deficiencies have an intermediate phenotype. They have a mixture of the two types of lesions and, after treatment with antimycobacterial agents, most of the granulomas are of the tuberculoid type. This again highlights the critical and nonredundant role of IFN-*γ*. However, the absence of IL-12 seems to be compensated in part by other factors, at least for cases of infection with weakly virulent mycobacteria [69, 70]. In any case, the study of this syndrome strongly highlighted the major role of granulomatous structures in the control of mycobacterial infections: genetic defects preventing granuloma formation are leading to disseminated infections even by poorly virulent mycobacteria, whereas genetic defects responsible for either delayed or poorly defined, yet maintained, granulomatous structures, drive to milder susceptibility to mycobacterial infections.

Other Th1-polarising cytokines, such as IL-27 (a member of the IL-12 family), have been described. As expected, the CD4^+^ T cells of mice with impaired IL-27 signalling produce lower levels of IFN-*γ*. However, this lower level of IFN-*γ* is, surprisingly, associated with a better control of bacterial growth. This is thought to reflect the regulatory effects of IFN-*γ*, decreasing lymphocyte survival. Consistent with this notion, IL-27R-deficient mouse granulomas contain larger numbers of lymphocytes than wild-type granulomas [71].

CD4^+^ T cells have been shown to have cytotoxic activity against *M. tuberculosis*-infected macrophages that is at least partly mediated by the FAS/FASL pathway, which would contribute to bacterial clearance [72–74]. The CD4^+^ T cells of patients with IFNGR-I deficiency have low levels of FAS expression and are impaired in their killing activity. This provides a mechanism by which IFN-*γ* could participate in the control of mycobacterial infection [72].

Gallegos et al. carried out a series of adoptive transfers of T cells with different genetic alterations in mice of various genetic backgrounds and found that IFN-*γ*-deficient CD4^+^ T cells controlled *M. tuberculosis *infection as efficiently, or almost as efficiently as wild-type cells when transferred into wild-type or IFN-*γ* KO backgrounds, respectively. This suggests that, at least in mice, IFN-*γ* is not critical for the effector function of Th1 cells [75].

In addition to the classical Th1 response, a Th17-type response is also triggered by mycobacterial infection. IL-17 is a proinflammatory cytokine driving the recruitment of effector cells, such as neutrophils, and participating in the activation of macrophages. Some IL-17-producing CD4^+^ T-cells are present in mycobacterial granulomas, but *γ*/*δ* T lymphocytes seem to be the chief producers of IL-17 in this context [76], as discussed below in the *γ*/*δ* section.

The CD4^+^CD25^+^FoxP3^+^ regulatory T-cell compartment is expanded both in patients with active TB [77] and in mice infected with Mtb [78]and these cells have also been shown to accumulate in lung granulomas. They limit the intensity of the immune response to the bacteria in a manner that seems to be independent of IL-10, as shown in depletion studies in mice and, *ex vivo,* in human PBMCs. They are thought to play an important role in the establishment of persistent infection.

#### 4.2.2. CD8^+^ T-Cells

Mice lacking CD8^+^ T cells (ß2m^−/−^ MHC-I-deficient mice [79–82] or animals depleted of CD8^+^ T cells [82]) are more susceptible to mycobacterial infection than wild-type animals. However, they have a less severe phenotype than CD4^+^ T cell-deficient mice. They display susceptibility to virulent mycobacteria and the infectious phenotype is dependent on the size of the inoculum [65, 79, 82].

In mouse lung granuloma, CD8^+^ T cells are initially found towards the periphery, migrating deeper into the structure as the disease progresses [8]. CD8^+^ T cell-deficient mice infected with *Mtb *form granulomas, but the functioning of these structures is impaired. They have marked central necrotic zones not seen in wild-type mice. This seems to be due to a lack of apoptosis induction in infected cells, resulting in the degeneration of these cells and an increase in neutrophil infiltration into the lesions [79, 83].

By immune reconstitution of athymic mice with IFN-*γ* ^−/−^ CD8^+^ T cells, Tascon et al. showed that this cytokine was involved in the role of CD8^+^ T cells in protecting against TB [84]. However, neither granzymes nor perforin seem to play a critical role in the anti-TB activity of CD8^+^ lymphocytes in mice [85, 86]. Cooper et al. observed no role of Fas (CD95)/FasL interaction [85], whereas Turner et al. found that, like CD8KO mice, FAS (and FASL) KO mice failed to control chronic Mtb infection and had displayed impaired granuloma formation, with higher levels of necrosis and neutrophil infiltration [83]. In humans, Mtb*-*specific CTL have been found [87] and shown to direct the granulysin-mediated lysis of the bacteria [88]. It has been suggested that CD8^+^ CTLs have predominantly antimycobacterial activity, whereas CD4^+^ CTLs (see above) have a more immunomodulatory role, in the removal of infected APCs [74].

Ordway et al. also suggested another role for CD8^+^ T cells in the granuloma. They found that, during chronic infection, activated CD8^+^ T cells produced the chemokine XCL1 (lymphotactin), which negatively regulates IFN-*γ* production by CD4^+^ T cells. This would contribute to the stability of the granuloma [68].

#### 4.2.3. *γ*/*δ* T Cells

*γ*/*δ* T cells are nonconventional T cells with a TCR composed of *γ* and *δ* chains. These cells are much less variable than *α*/*β* T cells and are considered to act as an intermediary between the innate and adaptive immune responses. There is some debate about their role in the antimycobacterial response in mice [80, 81, 89]. Nonetheless, the *γ*/*δ* T-cell compartment has been shown to expand after mycobacterial infection in mice [90], macaques [91] and humans [92]. This compartment also expands rapidly upon restimulation, in a reminiscent manner of memory cells [91, 92]. Several mycobacterial peptide and nonpeptide antigens seem to be recognized by *γ*/*δ* T cells [93].

These cells were first described in granulomas more than 20 years ago and seem to be involved in the formation and development of these structures [80, 94, 95]. In mice intraperitoneally infected with BCG, the recruitment of *γ*/*δ* T cells to inflammatory sites has been observed at early stages of infection [96]. Granulomas are present in similar numbers in *γδ*-KO and wild-type mice, but they are larger and less organised in the KO mice [80, 81, 89]. At high multiplicities of infection, *γδ*-KO granulomas display a massive influx of neutrophils. These changes are not related to bacterial multiplication, which does not differ between wild-type and *γ*/*δ*-KO mice. This suggests that *γ*/*δ* T cells may affect the organisation and inflammatory state of the granulomatous lesions [89].

Saunders et al. reported different results for *M. avium* infection. They found a lower influx of neutrophils and a lower level of necrosis in *γ*/*δ* KO mice infected with the 724 strain than in the wild type, whereas this pattern was not observed with the 2–151 strain. The authors suggested that *γ*/*δ* T cells stimulate macrophage influx into the tissue, but that with the 724 strain, a protective *α*/*β* T-cell response cannot be mounted. The infected macrophages therefore degenerate, resulting in high levels of inflammation and tissue damage. Conversely, in mice infected with the 2–151 strain of *M. avium *or with *M. tuberculosis,* a robust *α*/*β* T-cell response results in control [97] of the infection.

In mice, *γ*/*δ* T cells have been shown to be the main subset of T cells producing IL-17 in response to mycobacterial infection. This IL-17 production is dependent on antigenic stimulation and exposure to IL-23 [76, 98]. We have data indicating that *γ*/*δ* T cells are also the main producers of IL-17 in granulomas formed *in vitro* after the stimulation of human PBMCs with BCG (Deknuydt et al., in preparation). In a study of tissue from patients with BCG lymphadenitis, Kim et al. found that the *γ*/*δ* T cells were located principally at the periphery of granulomas with no necrotic zone [99]. However, Falini et al. found *γ*/*δ* T cells surrounding and within the necrotic zone of granulomas from patients with TB lymphadenitis [100]. Consistent with these findings, in a study of pulmonary tissue samples from TB patients, we found that only lesions with a caseous zone at the centre harboured *γ*/*δ* T cells, which were distributed in a ring around this zone (Deknuydt et al. in preparation).

#### 4.2.4. Natural Killer T (NKT) Cells

NKT cells, which express both an *α*/*β* TCR and NK cells markers, are like *γ*/*δ* T cells, at the interface of innate and acquired immunity. They recognise lipid ligands, such as glycosyl ceramides, presented by the MHC molecule CD1d. The subcutaneous injection of deproteinated mycobacterial cell wall into mice has been shown to induce granuloma-like structures with a high NKT-cell content, these T cells being activated by the recognition of bacterial PIMs [101].

However, granuloma formation in the lungs of NKT KO mice infected intranasally with Mtb seemed to be as efficient as that in wild-type mice [102]. Dieli et al. infected V*α*14 NKT cell-deficient mice with BCG via the intravenous route and found that these mice contained the infection as well as wild-type mice. However the V*α*14-NKT KO mice produced larger granulomas with a central necrotic zone not found in the wild-type mice and had higher levels of TNF-*α*. This suggests that NKT cells may play an anti-inflammatory role in the mycobacterial granuloma [103]. The treatment of Mtb-infected mice with *α*-galactosyl-ceramide, a potent activator of NKT cells, has been shown to decrease bacterial load and to improve the survival of the animals. The lung lesions of the treated mice were less necrotic and contained a larger number of lymphocytes [104]. Necrotic granulomas have been shown to express the *SapC* gene strongly. This gene encodes a protein involved in ceramide metabolism and in the transfer of mycobacterial lipid antigens from intralysosomal membranes to CD1d [105].

Further studies are required to determine the exact ligands of NKT cells and the effect of these cells on the immune response in the context of mycobacterial infection.

#### 4.2.5. B Lymphocytes

The response to mycobacterial infection is based mostly on cellular immunity, with the role of humoral immunity in protection against TB remains a matter of debate [106, 107]. Nonetheless, B cells have been implicated in the organisation and development of granulomatous lesions in the lung. In mice, B cells account for 1–10% of the leukocytes present in the granuloma [64], and their recruitment is dependent on the chemokine CXCL13 [108].

The lungs of mice with no B cells contain fewer granulomas than those of wild-type mice, and these granulomas are much smaller with little cellular infiltrate. However, they display higher levels of neutrophil and CD8^+^ T-cell recruitment. These changes are not correlated with differences in the ability to contain the infection, as the lungs of wild-type mice and mice with no B cells contain similar numbers of bacteria [108, 109]. An absence of B cells has also been shown to have no effect on tuberculosis progression during the chronic phase, over a period of 250 days [110]. B cells form aggregates in both mice [64, 108] and humans [64, 111]. These aggregates resemble the follicular structures of the lymph nodes. However, these B-cell clusters are associated principally with monocytes in mice and with T lymphocytes in humans. These structures are more frequently found in patients with latent tuberculosis and are thus associated with good immune control of the disease [112].

Cells of the B-1 subset are present in the peritoneal and pleural cavities. Their function remains unclear, but they may serve as a first line of defence, as described for *γ*/*δ* T cells. X-linked immunodeficient (Xid) mice lacking B-1 cells are more susceptible to BCG infection than their wild-type counterparts. Xid mice have disorganised granulomas with higher levels of macrophage influx and fewer T cells (particularly CD4^+^). Thus, B-1 cells appear to be involved in granuloma formation and the inflammatory state, at least through their downregulation of MCP-1 secretion. However, the presence of these cells within the granuloma has not been demonstrated [113]. B-1 cells have recently been shown to polarise anti-inflammatory macrophages resembling the AAMs found in tuberculous granulomas. This polarisation seems to be driven principally by IL-10 secretion [114].

#### 4.2.6. Necrosis

The granuloma tends to become necrotic in susceptible individuals, facilitating bacterial spread by coughing. The necrotic tissue has a characteristic “caseating” appearance [115, 116]. It is assumed that apoptosis kills the bacteria and promotes antigen presentation, thereby stimulating T cells, whereas the necrosis of infected cells releases the bacteria and promotes inflammation and tissue damage (see [117]). It remains unclear why some individuals have necrotic lesions whilst others do not progress beyond latent infection, and the factors triggering necrosis have yet to be identified. The tuberculous granulomas of mice induced by commonly used strains do not display caseous necrosis, whereas this feature is observed in other animal models, such as guinea pigs, rabbits, and macaques, and in mice infected with certain strains of *M. avium*. However, the recently described Nos 2^−/−^ mice model that develop necrotic granulomas similar to those of humans [22], as well as the use of C3HeB/FeJ mice that naturally develop lesions with liquefactive necrosis upon Mtb infection [23, 24], may represent more relevant mice models and thus very useful tools to study the bacterial/cell interplay, and for drug development. Caseous necrosis is associated with a hypoxic state of the lesions [64, 118, 119]. In the model of *M. avium *infection in mice, Aly et al. showed that IFN-*γ* and reduced vascularisation/hypoxia of the lesion were involved in the caseation process [120]. In humans, transcriptional analyses of microdissected tuberculous granulomas have shown that caseation is associated with an increase in lipid metabolism. This is consistent with a role for foamy macrophages in the formation of the central necrotic zone [35, 105].

There are good reasons to think that neutrophils also play a role in necrosis. It remains unclear whether these cells are protective or damaging, but, when Mtb-infected animals are repeatedly challenged with mycobacterial antigens, the lesions become necrotic and contain a higher proportion of granulocytes [121].

#### 4.2.7. Imaging Technologies

Early diagnosis and appropriate treatment of TB are important, to reduce transmission and favour the elimination of the bacterium. Tuberculosis is diagnosed on the basis of laboratory tests, chest X-rays, CT/MRI scans, microbiologic smears, and cultures of body fluids, including sputum in some cases, histological analysis, and biopsy. Diagnosis and follow up have recently been improved by the development of imaging techniques using radiopharmaceutical compounds. FDG-PET can be used not only for diagnosis, but also for monitoring throughout tuberculosis treatments, particularly in cases of multidrug-resistant infections. Soussan et al. identified two different patterns of pulmonary TB by FDG-PET/CT [122]. Harper et al. used PET to describe the granulomas of C3HeB/FeJ mice [23]. Sathekge et al. recently published a review in which they summarize the various technologies used for the diagnosis of TB and for monitoring the response to antimycobacterial treatment. In this review, they pointed out that one of the key problems in tuberculosis diagnosis is the invasive nature of the methods used [123]. The development of new, safe and noninvasive methods for imaging is therefore likely to prove highly beneficial.

## 5. Conclusions

We now have various experimental models that should help to unveil the mysteries of the complex host-pathogen relationships taking place in the mycobacterial granuloma. Granuloma formation seems to be primarily a host-defence mechanism for containing the bacteria, but it also shelters the bacteria, providing them with a niche in which they can persist in a latent form until an opportunity arises for re-activation and spread. An understanding of the physiopathology of granulomas is critical for the design of new vaccines and antituberculous drugs.

Granulomas are not restricted to mycobacterial infections, being found in many different kinds of bacterial, fungal or viral infections, and even in noninfectious inflammatory diseases [4]. Thus, the knowledge obtained about mycobacterial granulomas and some of the models used to study them may be useful in the fight against other diseases.

## Figures and Tables

**Table 1:** Models for studying Mtb infection and the granulomatous response.

Model	Advantages	Drawbacks
Monkey	Granuloma similar to humans.	Difficult to handle.
		Expensive.

Guinea pigs/rabbits	Granuloma similar to humans.	Restricted availability of reagents.
	Easy to handle.	Genetic manipulation difficult.

Mice	Easy to handle.	Granulomas often differ in many ways from
	Model of choice for genetic studies.	human granulomas (e.g., cellular composition and progression to necrosis).

Zebrafish embryo	Easy to handle.	*M. marinum* rather than *M. tuberculosis* infection.
	Good for real-time experiments and live imaging (the larvae are transparent).	
	Good for studies of the initial steps of granuloma formation and the role of innate immunity.	No lymphocytes present in the embryo.

*In v* *it* *ro* granuloma formation from human PBMCs	Mimics the physiological granuloma.	Some important elements present in the lung compartment but not in PBMCs may be lacking.
	Possible to study the early steps of granuloma formation.	
	Flexible (e.g., use of various strains of bacteria, easy addition of cells, cytokines, drugs).	

*In s* *il* *ic* *o* modelling of granuloma formation	Not expensive.	Highly dependent on the initial parameter settings and cannot take previously unknown information into account.
	Study of the early steps of granuloma formation possible.	
	Flexible.	

**Table 2:** Main chemokines and cytokines involved in the granulomatous response.

Chemokines/cytokines	Main producers	Targets/role
CXCL8 (IL-8)	Alveolar macrophages.	Recruitment of neutrophils.
	Epithelial cells of the lung.	

CCL2 (MCP-1)	Monocytes and alveolar macrophages.	Recruitment of macrophages and other immune cells.

CCL3 (MIP-1a), CCL4 (MIP-1b) CCL5 (RANTES)	Alveolar macrophages.	Recruitment of macrophages and other immune cells.

CXCL9, CXCL10 (IP-10), CXCL11	Bronchial epithelial cells.	Recruitment of immune cells.

CCL19/CCL21	Stromal cells of the lymph nodes.	Recruitment and priming of IFN-*γ*-producing T cells.
		Migration of DC from the lung to draining lymph nodes.

CXCL13	Dendritic cells, stromal cells of the lymph nodes.	Recruitment of B cells and formation of the granuloma-associated follicular structures.

IL-12/IL-23	Dendritic cells, macrophages.	Th1 polarisation of CD4^+^ T cells.

IFN-*γ*	CD4^+^ (Th1) and CD8^+^ T cells, NK.	Activation of macrophages.
		Induction of NO synthesis and bacterial killing.

TNF-*α*	CD4^+^ T cells (Th1), macrophages.	Proinflammatory.
		Induction of chemokine production.
		Activation of macrophages.
		Critical for granuloma formation.

IL-1	Macrophages, DCs.	Proinflammatory.
		Recruitment and activation of phagocytes.

IL-17	LT *γ*/*δ*, CD4^+^ T cells (Th17).	Proinflammatory.
		Involved in neutrophil recruitment and macrophage activation.

IL-10	Tregs, B-1 cells, AAM.	Anti-inflammatory.
		Polarisation of macrophages towards the AAM type.

TGF-*β*	Tregs, AAM.	Anti-inflammatory.
